# Guidance for dysmorphic sacrum fixation with upper sacroiliac screw based on imaging anatomy study: techniques and indications

**DOI:** 10.1186/s12891-023-06655-9

**Published:** 2023-06-30

**Authors:** Tan Shan, Li Hanqing, Ai Qiuchi, Xing Junchao, Xu Meitao, Gao Shichang, Hou Tianyong

**Affiliations:** 1 Department of Orthopedics, the First Affiliated Hospital of Army Medical University, Chongqing, China; 2 Department of Orthopedics, The First Affiliated Hospital of Chongqing Medical University, Chongqing, China

**Keywords:** Dysmorphic sacrum, Sacroiliac screw, Imaging anatomy, Sacral fracture

## Abstract

**Objective:**

This study aimed to investigate the techniques and indications of upper sacroiliac screw fixation for the dysmorphic sacrum.

**Methods:**

The dysmorphic sacra were selected from 267 three-dimensional pelvic models. The dysmorphic sacra which couldn’t accommodate a 7.3 mm upper trans ilio-sacroiliac screw were classified as the main dysmorphic sacra. Then, the size of the bone corridor, the length of the screw in the corridor, and the orientation of the screw were measured. The insertion point on the sacrum was identified by two bone landmarks.

**Results:**

totally, 30.3% of sacra were identified as the main dysmorphic sacra. The inclinations of the screw oriented from posterior to anterior were (21.80 ± 3.56)° for males and (19.97 ± 3.02)° for females (p < 0.001), and from caudal to cranial were (29.97 ± 5.38)° for males and (28.15 ± 6.21)° for females (p = 0.047). The min diameters of the corridor were (16.31 ± 2.40) mm for males and (15.07 ± 1.58) mm for females (p < 0.001). The lengths of the screw in the Denis III zone were (14.41 ± 4.40) mm for males and (14.09 ± 5.04) mm for females (p = 0.665), and in the Denis II+III zones were (36.25 ± 3.40) mm for males and (38.04 ± 4.60) mm for females (p = 0.005). The rates of LP-PSIS/LAIIS-PSIS were (0.36 ± 0.04) for males and (0.32 ± 0.03) for females (t = 4.943, p < 0.001). The lengths of LPM were (8.81 ± 5.88) for males and (-4.13 ± 6.33) for females (t = 13.434, p < 0.001).

**Conclusion:**

When the sacrum has the features of “sacrum not recessed” and/or “acute alar slope”, the conventional trans ilio-sacroiliac screw couldn’t be placed safely. The inclination oriented from posterior to anterior and from caudal to cranial are approximately 20° and 30°, respectively. The bone insertion point locates in the rear third of the anterior inferior iliac spine to the posterior superior iliac spine. The sacroiliac screw is not recommended to fix the fractures in Denis III zone.

## Introduction

The posterior pelvic ring is the main structure of weight-bearing, therefore, anatomical reduction and rigid fixation of the posterior ring are the main purposes of the surgical management for unstable pelvic ring traumatic injures. Percutaneous sacroiliac (SI) screw fixation has been a safe and reproducible method for sacroiliac dislocation and sacral fracture [1, 2], however, it is technically demanding, and the surgeon must have an thorough understanding of sacral osteology as well as fluoroscopic images [3, 4]. As reported, the upper sacral dysmorphism causes a significant increase of malposition of the SI screw, resulting in the decrease of pelvic ring stability and the increase of neurovascular injures with a risk of incidence as high as 18% [4–6]. Even in certain conditions, one of the contraindication of SI screw fixation is the upper sacral dysmorphism [7]. Although the introduction of surgical navigation systems has made SI screw insertion safer and more accessible, conventional fluoroscopy is the standard technique in most hospitals [4, 7–9]. There are several studies about the safe placement of the upper SI screw, but few quantitative studies have been done for the dysmorphic sacra [4, 10–12]. The aims of our study are to prevent injury to neurovascular structures caused by the malposition of screw, reduce the radiation exposure time and improve the clinical results in patients. The correct insertion point and orientation are the prerequisites to ensure the safe and quick placement of SI screws [13]. Therefore, in our study, the computer tomography (CT) scans data of pelvis were used to reconstruct the three-dimensional (3D) project, then the virtual screws were placed in the upper sacral segment, thus the insertion point, orientations and indications of the screw were identified reversely.

## Materials and methods

### Specimens collection

**Fig. 1:**
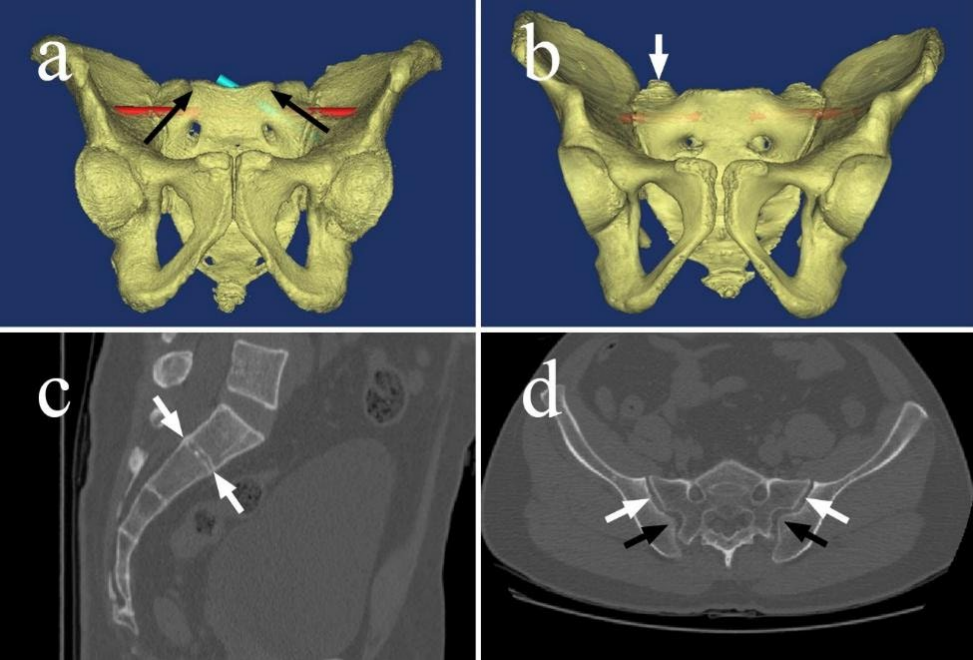
The dysmorphic characteristics of upper sacral segment. (**a**) The TISI screw (red) couldn’t be completely intraosseous placed in the main dysmorphic pelvis, but the oblique screw (green) could be. The upper sacral segment was not recessed in the pelvis. The black arrows oriented from lateral-inferior to middle-superior showed the acute alar slope. The upper sacral neural foramina were not circle-shaped. (**b**) The TISI screw (red) could be intraosseously placed safely in the minor dysmorphic pelvis. The upper sacral segment was recessed in the pelvis. The white arrow pointed out the mammillary process. The upper sacral neural foramina were circle-shaped. (**c**) The residual disc between the first and second sacral segments could be seen on the CT scan clearly. (**d**) “tongue-in-groove” sacroiliac morphology: the bilateral anterior parts of the sacroiliac joint were parallel (white arrows), however, the posterior parts formed an interlocking pattern (black arrows)

All experimental protocols were approved by the Ethics Committee of the First Affiliated Hospital of Chongqing Medical University. 267 healthy adult pelvises of 64-slice spiral CT scans data were collected from January 2018 to January 2020 in the Medical Imaging Department. The pelvic 3D projects were reconstructed by the Materialize’s interactive medical image control system (Mimics) 16.0 software based on CT data. These dysmorphic sacra were identified according to the six qualitative characteristics [2, 14] as followings: (a) an upper sacral segment not recessed in the pelvis (sacrum not recessed, for short), (b) an acute alar slope, (c) the presence of mammillary processes, (d) a residual disc between the first and second sacral segments, (e) dysmorphic upper sacral neural foramina, (f) “tongue-in-groove” sacroiliac morphology. Now, these characteristics were easier to be identified on the 3D model and CT scan (Fig. 1). As several parties proposed that, for the dysmorphic sacra, the trans ilio-sacroiliac (TISI) screw could not be completely intraosseous inserted [4, 10–12, 15–17]. In our study, the dysmorphic sacra were tested as Gardner reported by a 7.3 mm TISI screw simulated by a virtual cylinder. According to the results, these sacra were divided into two groups: (a) main dysmorphic sacra : the TISI screw could not be placed safely (Fig. 1a); (b) minor dysmorphic sacra: the TISI screw could be placed safely (Fig. 1b).

### Quantitative measurement of the upper sacral corridor

**Fig. 2:**
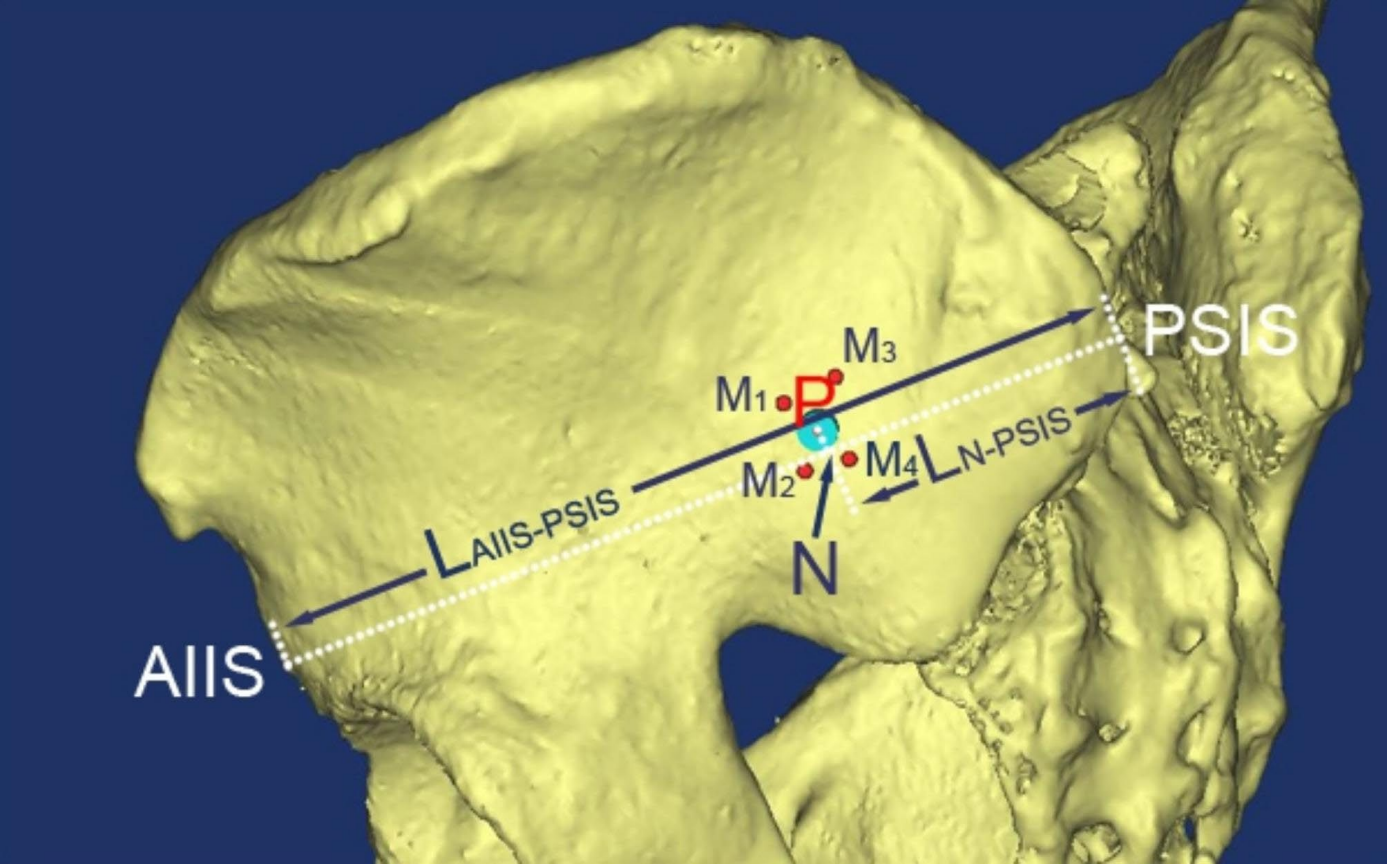
Location of the insertion point on the ilium surface

Four screws were placed in the upper sacral pedicle and were obliqued to its axis. The four screws were tangent with the anterior-superior, anterior-inferior, posterior-superior and posterior-inferior cortex of the upper sacral pedicle, respectively, and were intersected at points M1, M2, M3 and M4 on the lateral cortex of ilium (Fig. 2). The gravity of the quadrilateral M1-4, which represents its center, was chosen as the insertion point P.

Two assisted bone landmarks, anterior inferior iliac spine (AIIS) and posterior superior iliac spine (PSIS), were assisted to locate the insertion point on the ilium surface. As Fig. 2 showed, the distance from AIIS to PSIS was measured as LAIIS-PSIS. Line PN was perpendicular to line AIIS-PSIS and the distance from point N to PSIS was measured as LN-PSIS. If the P was located above the line AIIS-PSIS, the length of PN (LPN) was marked as plus, otherwise, marked as minus.

**Fig. 3:**
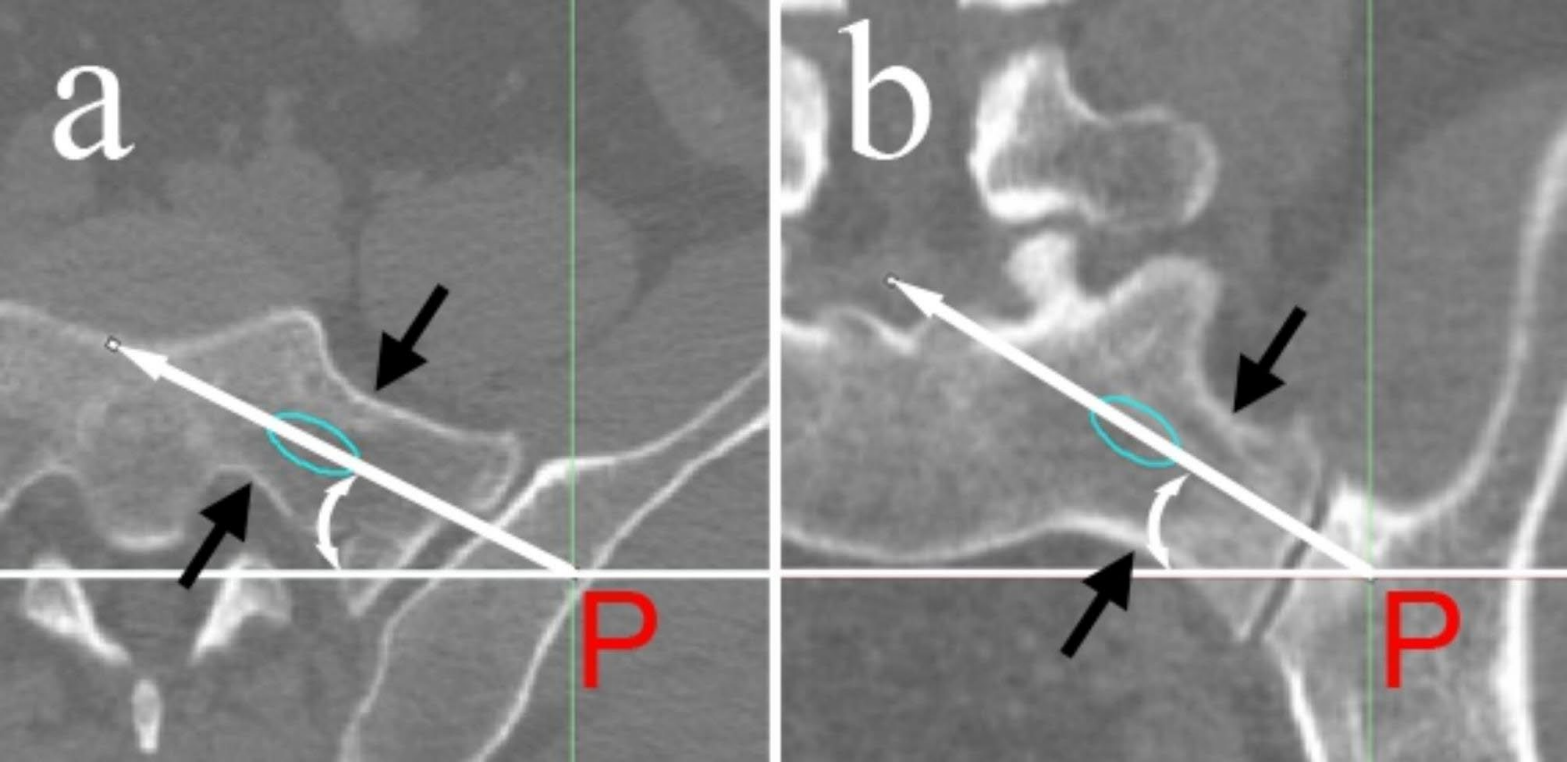
Measurement of the inclination angles of the upper SI screw. The green ellipse represented the screenshot of the screw on the CT scan, and the long white arrows represented its axis directions. (**a**) On the axial scan, the two short black arrows pointed out the nearest anterior and posterior cortical bone to the screw. (**b**) On the coronal scan, the two black arrows pointed out the nearest superior and inferior cortical bone to the screw

To find out the best inclination angles, the tail of the screw was fixed at P. On the coronal scan, the tip of the screw was rotated around the insertion point until it was tangent with the nearest superior or inferior cortical bone of the upper sacral pedicle (Fig. 3a). Thus, the max and min cranial angles were measured. The best cranial angle was calculated as the mean value of the max and min angles. A similar procedure was done on the axial scan to get the anteversion angle (Fig. 3b). The tip of the screw was rotated until it was tangent with the nearest anterior or posterior cortical bone of the upper sacral pedicle. The inclination angles were subtended by a line drawn parallel to the axis of the screw and the horizontal line. Then, according to the insertion point and best inclination angles, we inferred that the screw was placed in the relative center of the upper sacral pedicle.

**Fig. 4:**
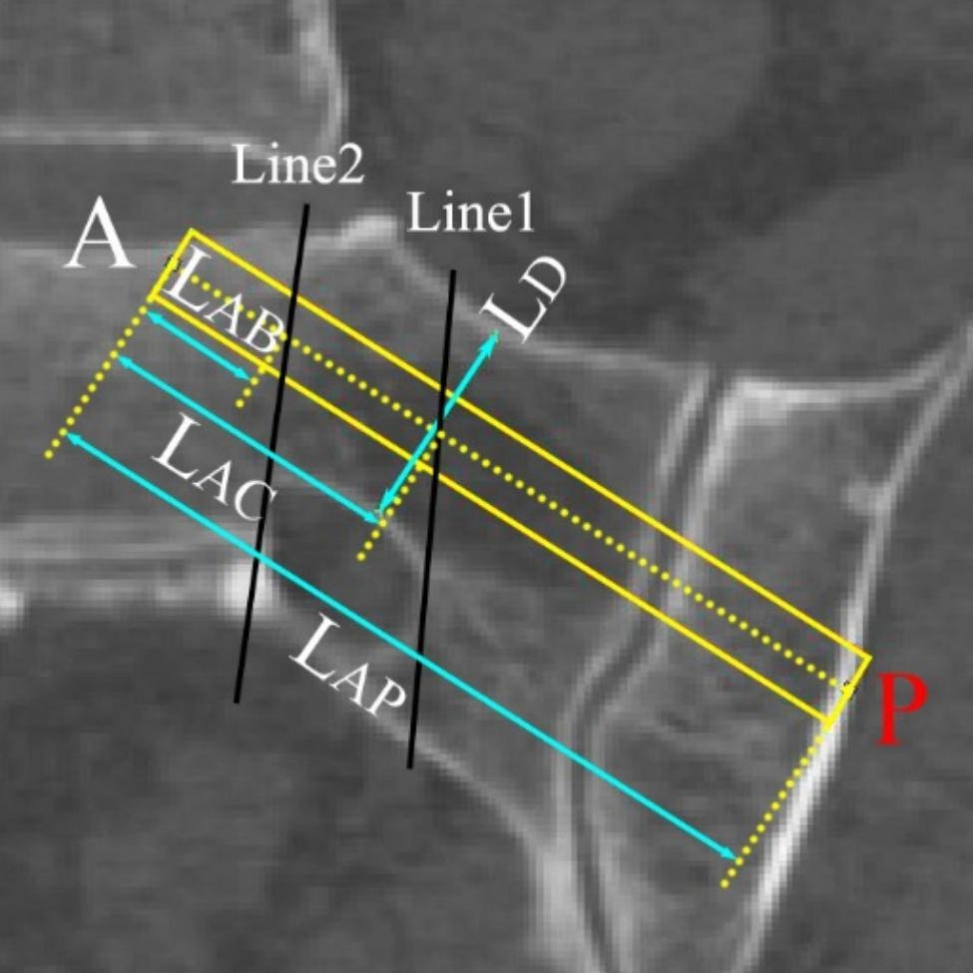
Measurement of the relevant parameters of the upper sacral pedicle. In the Fig. 4, the two black lines divided the sacrum into Denis I, II and III zones

As reported, in the upper sacral pedicle, the superior-inferior diameter was smaller than the anterior-posterior diameter [18, 19]. Therefore, on the coronal scan, the width of the narrowest part of the pedicle was measured as the min diameter (LD) of the upper sacral pedicle (Fig. 4). The length of the screw in Denis III zone (LAB), Denis II+III zones (LAC) as well as the full length (LAP) were measured.

### Statistical analysis

SPSS 19.0 software was chosen for the analysis. The difference between rates was tested by chi-square test. Measurement data were expressed in mean differences ± standard deviation and analyzed by the independent sample t-test. p < 0.05 was required for a statistical significance.

## Results

### Dysmorphic statistics

**Fig. 5:**
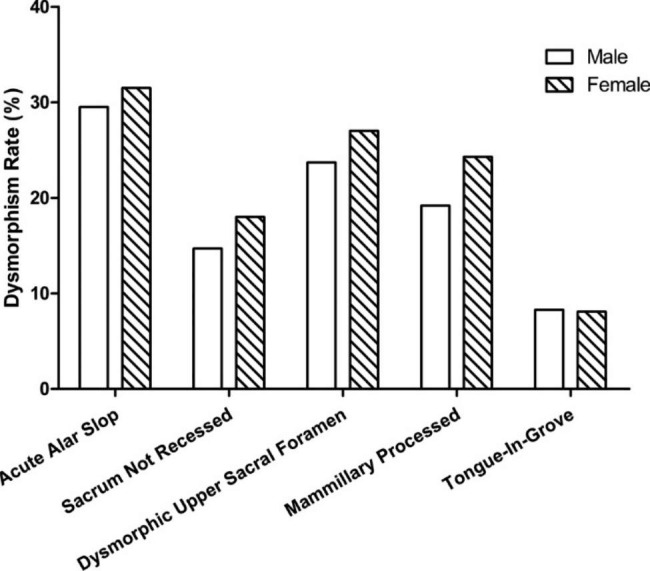
Sex-specific prevalence of the signs of sacral dysmorphism

There were 156 males and 111 females with a median age of 50.1 years (range, 18-90y). The “residual disc” was identified in 98.1% of all samples (males: 98.1%, females: 98.2%, *x*^*2*^ = 0.005, *p* = 0.943) on CT scan. Therefore, in the following paper, “residual disc” was not regarded as a dysmorphic characteristic anymore. Except for the “residual disc”, 39.3% of the dysmorphic sacra (males: 34.6%, females: 45.9%, *x*^*2*^ = 3.489, *p* = 0.062) were selected from all subjects. The “acute alar slop” was the most frequent detected sign, followed by “dysmorphic upper sacral foramen”, “mammillary processed”, “sacrum not recessed” and “tongue-in-grove” (Fig. 5). The sex-specific differences of dysmorphism rates for all characteristics were not statistically significant.

We discovered an interesting thing. For the main dysmorphic sacra, there must exist the characteristics of “sacrum not recessed” and/or “acute alar slop”, however, the minor dysmorphic sacra didn’t have the two characteristics. Therefore, the “sacrum not recessed” and “acute alar slop” were classified as “main dysmorphism”, and the other three characteristics were classified as “minor dysmorphism”. We inferred that the safe placement of the upper SI screw for dysmorphic sacrum was only influenced by the “main dysmorphism”, and the “minor dysmorphism” were less mentioned in the following.

The main dysmorphic sacra included 81 specimens, and accounted for 30.3% of all specimens. The minor dysmorphic sacra, which included 24 specimens, and accounted for 9.0% of all specimens. The “main dysmorphism” often coexisted with “minor dysmorphism” (Table 1), while the “minor dysmorphism” could exist alone. The number of lonely existed “tongue-in-groove” was 11 (males:5, females:6), the “mammillary process” was 7 (males:1, females:6) and the “dysmorphic upper sacral foramen” was 6 (males:2, females:4).

**Table 1:** Cumulative prevalence number of the combination types of main and minor dysmorphism

	The number of minor dysmorphism characteristics							
	None	One			Two			Three
		①	②	③	①+②	①+③	②+③	①+②+③
Main dysmorphism	3	23	16	0	28	4	2	5

①: dysmorphic upper sacral foramen, ②: mammillary process, ③: tongue-in-groove

### The orientation of the screw

The anteversion inclination angles of the screw were (21.80 ± 3.56)° for males and (19.97 ± 3.31)°for females (*t* = 3.901, *p* < 0.001). The cranial inclination angle were (29.97 ± 5.38) °for males and (28.15 ± 6.21)°for females (*t* = 1.998, *p* = 0.047).

### Several parameters of the bone corridor

The min diameter of the corridor were (16.31 ± 2.40) mm for males and (15.07 ± 1.58) mm for females (*t* = 3.750, *p* < 0.001). The lengths of the screw in different Denis zones were shown in Table 2. The lengths of the screw in Denis III zone for almost 70% of samples were no longer than 16 mm, and 100% were shorter than 32 mm. The length of the screw in Denis II+III zones for almost 90% of samples were longer than 32 mm and 100% were longer than 16 mm.

**Table 2:** The lengths of the screw in different parts of the bone corridor (Mean ± SD, mm)

Bone corridor	Males	Females	*t*	*p*
Denis III	14.41 ± 4.40	14.09 ± 5.04	0.438	0.662
Denis II+ III	36.25 ± 3.40	38.04 ± 4.60	-2.838	0.005
Full	80.13 ± 5.14	80.13 ± 5.14	1.108	0.270

### The location of the insertion point on the ilium surface

The distance from P and AIIS to PSIS as well as the distance from P to line AIIS-PSIS were shown in Table 3. The location of the insertion point was estimated by the two bone landmarks and was described by the ratio of L_P-PSIS_/L_AIIS-PSIS_ as well as L_PM_. The rates of L_P-PSIS_/L_AIIS-PSIS_ were (0.36 ± 0.04) for males and (0.32 ± 0.03) for females (*t* = 4.943, *p* < 0.001).

**Table 3:** The location relationship between the insertion point and bone landmarks (Mean ± SD, mm)

Length	Males	Females	*t*	*p*
L_P−PSIS_	55.22 ± 8.00	49.62 ± 6.05	4.885	< 0.001
L_AIIS−PSIS_	156.97 ± 9.49	153.74 ± 8.26	2.27	0.025
L_PM_	8.81 ± 5.88	-4.13 ± 6.33	13.434	< 0.001

## Discussion

### Dysmorphic sacra

In the early 1990s, the percutaneous fixation via SI screws began to be used to stabilize posterior pelvic ring, sacral fractures and sacroiliac joint injuries. Compared with the classical method, this new method decreases operating time, soft tissue injury, blood loss, and infection risk [20]. However, the incidence of screw malposition is 3-25% [21, 22], and the rate of neurological damage has approached to 18% [14, 23, 24], as a result of complex pelvic osteology and especially sacral dysmorphism.

In our study, the “residual disc” could be seen on CT scans for almost samples (98.1%). Weigelt found that the “residual disc” was the most frequent detected sign which present in 70% of all patients [25]. Miller had reported that the “residual disc” could show in persons with non-dysmorphic sacral anatomy [19]. Above all, the “residual disc” was not regarded as a dysmorphic characteristic in our study. In 1996, Routt et al. identified six characteristics of upper sacral dysmorphism in 35% of cases (males: 30%, females: 41%) by x-ray [2]. Compared with the results of Routt, although excluded “residual disc”, the 39.3% dysmorphism rate (males: 34.6%, females: 45.9%) in our study was still higher. Wu studied another four categories of dysmorphic sacra: accessory auricular surface, sacral skewness, transitional vertebra and sacral spina bifida occulta, and the overall rate of variations was 58.1% (males: 57.4%, females: 59.5%) [26]. Hasenboehler et al. only found 14.5% dysmorphic sacra (males: 12.2%, females: 19.2%), which include four dysmorphic types: increased alar slope, obliquity of the residual transverse process on the sacral ala, anomaly of the first sacral anterior neural foramina, and sacralized L5 or lumbarized S1 vertebrae [18]. In our opinion, the classification of Routt was more conducive to guide the safe placement of the upper SI screws in clinical.

Although, in the classification of Routt, there are six dysmorphic characteristics, only “sacrum not recessed” and “acute alar slope” complicate the safe placement of SI screws much. The alar slope is more acute in these patients, and it is associated with a notable inferior-lateral-posterior to superior-middle-anterior orientation. This oblique dysmorphic alar osteology makes TISI screw fixation impossible. Both the two characteristics often show on one sacrum. This phenomenon may be conducted by: in the growth and development of our body, the middle part of the sacral ala develops with the rising sacral body, however the lateral part is still connected with the inferior sacroiliac joint. As the rising of the upper sacral body, the round foramen may be stretched non-circular. The mammillary processes are deformed or under developed residual transverse processes from the sacralized L5 vertebral body. Above all, the appearance of the main dysmorphism often combines with minor dysmorphism.

Since the lumbosacral junction is one of the most variable regions of the spinal column [27], Miller thought when the sacrum fuses excessively cranially, creating sacralized L5 [19]. Therefore, the “sacrum not recessed” might be the representation of the “transitional vertebra”. Due to the different conditions of patients in our study, it is difficult to get the whole spin CT scans to judge whether the upper sacrum is the transitional vertebra.

### Indications of upper SI screw fixation for dysmorphic sacrum

For non-dysmorphic sacrum, indications for upper SI screw fixation include complete sacral fractures, sacroiliac joint disruptions, and combinations of these posterior pelvic injuries followed reduction. Incomplete sacral fractures and sacroiliac joint disruptions that contribute to pelvic ring instability may be addressed with SI screw fixation [19].

In 1988, Denis [28] classified the sacrum into three zones: the region of the alar (zone I), the sacral foramina (zone II), and the central sacral canal (zone III). As we all know that, only when all the screw thread pass the fracture line, the fracture fragments could be rigidly fixed. The thread length of the 7.3 mm pull screw was generally 16 or 32 mm. In our study, the length of the screw in Denis III zone was shorter than 32 mm for all subjects and shorter than 16 mm for 70% of patients (Table 2). The lengths in Denis II+III zones for 90% of patients were longer than 32 mm and 100% exceed 16 mm (Table 2). Therefore, the fractures in the Denis III zone could not be rigidly fixed by the upper SI screw, however, they could be treated by classical methods such as posterior planting and sacral bar. On the other hand, as reported [1, 29], patients with a dysmorphic sacrum typically have a safe zone at the second sacral segment that can accept a TISI screw. For these patients, the second sacral segment almost always provides a larger safe zone for screw insertion than does the upper sacral segment.

### Screw fixation technique for dysmorphic sacrum

Although the SI screw malposition rate is the lowest in the computer-assisted navigation techniques, conventional fluoroscopy is easier to get in most hospitals [4, 7–9]. Before the operation, good-quality plain pelvic radiographs and CT scans could be used to help the doctor to identify the dysmorphic sacrum and to estimate the orientation of the upper vertebral pedicle. Kellam recommend that CT scan reformatting parallel to the S1 superior end plate increases the likelihood of identifying a safe corridor for a TISI screw, especially in patients with evidence of sacral dysplasia [4]. As Chung reported that if the elevated height exceeded 13 mm, the pelvis was assigned to sacral dysmorphism and thus, could not apply the TISI screw fixation into S1 [11]. In our study, the inclination of upper SI screw for dysmorphic sacrum oriented from posterior to anterior on lateral view was about 20°, and from caudal to cranial on anterior-posterior view was about 30°. As the existence of anterior and cranial inclination angles, the surgeon must know that the insertion point on the skin was more caudal and more posterior than non-dysmorphic sacra. The mean rates of L_P-PSIS_/L_AIIS-PSIS_ were 0.36 for males and 0.32 for females (Table 3). The mean values of L_PM_ were 8.81 mm for males and − 4.13 mm for females (Table 3). Therefore, we infer that the insertion point on the bone surface was nearly located at the rear third of the connecting line from AIIS to PSIS. During the inserting procedure, in the fluoroscopic inlet view, the screw was located in the sacral alar and just posterior to the upper nerve tunnel. In the fluoroscopic outlet view, the screw was located in the sacral alar and just superior to the upper nerve tunnel [19]. What needs special attention was that, in the true lateral sacral view, the tip of the screw lies just cranial-anterior to the iliac cortical density (ICD) line for the main dysmorphic sacra [19]. However, in the non-dysmorphic and minor dysmorphic sacra, the ICD line is coplanar with the anterior sacral alar cortical bone, so the tip of the screw must lies caudal-posterior to the ICD line. Koning had reported that 37% of men and 34% of women don not have a complete osseous corridor to safely pass an 8 mm TISI screw across the S1 vertebral body [30]. In Mendel’s study, 20% of the 125 pelvises did not show a sufficient S1 corridor for a 7.3 mm screw [29]. In our study, the min diameters of the corridor were (16.31 ± 2.40) mm for males and (15.07 ± 1.58) mm for females. The full length of screw were (80.13 ± 5.14) mm for males and (80.13 ± 5.14) mm for females (Table 2). Therefore, no more than two 7.3*80 mm SI screws were recommended to be placed in the upper sacral segment.

### Summary

Non- and dysmorphic sacrum have distinct anatomic differences that must be noted prior to surgical intervention, especially the characteristics of “sacrum not recessed” and “acute alar slope”. For these patients, the skin insertion point which was more posterior and inferior than non-dysmorphic sacrum and the bone insertion point was located nearly the rear third of line AIIS to PSIS. The inclination oriented from posterior to anterior was about 20°, and that from caudal to cranial was about 30°. What needs specially attention was that the upper SI screw was not recommended to fix the fractures in Denis III zones. The correct insertion point and orientation could help to reduce the rate of malposition, reduce the radiation exposure time and improve the clinical results of patients.

## Data Availability

The datasets generated and/or analysed during the current study are not publicly available due to individual privacy of participants but are available from the corresponding author on reasonable request.

## Abbreviations

SI: sacroiliac
CT: computer tomography
3D: three-dimensional
TISI: trans ilio-sacroiliac
AIIS: anterior inferior iliac spine
PSIS: posterior superior iliac spine
ICD: iliac cortical density
